# Comparison and Analysis of Geometric Correction Models of Spaceborne SAR

**DOI:** 10.3390/s16070973

**Published:** 2016-06-25

**Authors:** Weihao Jiang, Anxi Yu, Zhen Dong, Qingsong Wang

**Affiliations:** 1 College of Electronic Science and Engineering, National University of Defense Technology, Changsha 410073, China; jiangweihao14@nudt.edu.cn (W.J.); dongzhen@nudt.edu.cn (Z.D.); 2 Equipment Academy of the Rocket Force, Beijing 100085, China; yilingsql@126.com

**Keywords:** spaceborne SAR, geometric correction, Range-Doppler, rational polynomial, polynomial, elevation

## Abstract

Following the development of synthetic aperture radar (SAR), SAR images have become increasingly common. Many researchers have conducted large studies on geolocation models, but little work has been conducted on the available models for the geometric correction of SAR images of different terrain. To address the terrain issue, four different models were compared and are described in this paper: a rigorous range-doppler (RD) model, a rational polynomial coefficients (RPC) model, a revised polynomial (PM) model and an elevation derivation (EDM) model. The results of comparisons of the geolocation capabilities of the models show that a proper model for a SAR image of a specific terrain can be determined. A solution table was obtained to recommend a suitable model for users. Three TerraSAR-X images, two ALOS-PALSAR images and one Envisat-ASAR image were used for the experiment, including flat terrain and mountain terrain SAR images as well as two large area images. Geolocation accuracies of the models for different terrain SAR images were computed and analyzed. The comparisons of the models show that the RD model was accurate but was the least efficient; therefore, it is not the ideal model for real-time implementations. The RPC model is sufficiently accurate and efficient for the geometric correction of SAR images of flat terrain, whose precision is below 0.001 pixels. The EDM model is suitable for the geolocation of SAR images of mountainous terrain, and its precision can reach 0.007 pixels. Although the PM model does not produce results as precise as the other models, its efficiency is excellent and its potential should not be underestimated. With respect to the geometric correction of SAR images over large areas, the EDM model has higher accuracy under one pixel, whereas the RPC model consumes one third of the time of the EDM model.

## 1. Introduction

With the development of synthetic aperture radar (SAR), SAR images play an increasingly important role in military reconnaissance and national economies. Because a SAR image records the back-scattering performance of surface features influenced by system survey precision and terrain undulations, SAR imagery contains several types of geometric distortion that seriously restrict their application. To effectively use a SAR image, precise geolocation of image pixels is required and is also necessary for precise geometric correction during later image processing.

In 1982, Curlander first proposed the use of satellite ephemeris, echo time delay and Doppler data to derive the absolute location of a target, a method called the Range-Doppler (RD) algorithm [1,2]. Some researchers then made improvements to the RD algorithm and analyzed the sources of location errors. In 2006, Wang et al. used polynomial fitting of the Doppler center frequency to adjust the Doppler equation, based on the phenomenon that Doppler center frequency does not equal zero, which produced a model that suits both spaceborne and airborne sensors [3]. In 2008, Fu et al. corrected the Earth model and developed a formula that added the target into the Earth radius to give the logical Earth model [4]. Many other scholars are conducting correlation studies.

The rational polynomial coefficients (RPC) model is a recently developed pattern geolocation model for SAR imagery. In 2001, Tao divided the rational function model into two sub-models, “terrain-dependent” and “terrain-independent”, based on the solution mode of the rational function coefficient [5]. In 2003, Di et al. compared the rectification results of the RPC model with the physical sensor model and discussed the feasibility of transformation from the RPC model to a physical sensor model [6]. In 2006, Fraser et al. systematically studied how to use the physical model to calculate the RPC model, introduced the solution for deviation compensation of elements of exterior orientation, and summed up the regulation of error propagation [7]. In 2006, Qin et al. first applied the RPC model to the geolocation of ERS SAR images, analyzed the precision of RPC coefficients for nine types, and determined the effect of the size of a control grid and slice numbers [8]. In 2008, Zhang et al. introduced the RPC model, based on the RD model, for the geometric rectification of TerraSAR-X and COSMO SkyMed high resolution SAR images, derived the rational function coefficients of the RPC model, and obtained the orthorectified images [9,10]. Some researchers improved the RPC model. Fraser et al. performed bias compensation on the RPC model [11], and Eftekhari et al. used a refined RPC model on TerraSAR-X images [12]. Liu used control points to refine the RPC model, proving a new geometric correction method for SAR images based on the RPC model [13]. Currently, the RPC model is increasingly being applied because of its high efficiency, ease of use and confidentiality.

The polynomial model is a traditional correction method, used for the orthorectification of optical images. It was used in the SAR imagery geometric processing during the initial development of SAR geometric correction theory. The polynomial model makes direct mathematical simulations of image distortion, avoiding the imaging space geometric process, which is simple and efficient. Some researchers studied the influence of elevation on geolocation error, providing a refined polynomial method for projection differences, which improved geolocation results [14,15]. On the basis of the Range-Doppler location model, a high-precision geometric correction method supported by an a priori DEM was proposed by Wang et al. [16]. The method was named the elevation derivation (EDM) model; it uses interpolation fitting instead of an iterative process and has high efficiency and high precision. Yu et al. used TerraSAR-X data in experiments [17] that showed that the new method can significantly improve the geolocation accuracy of SAR imagery and decrease the processing time by ten times under the ignorable error compared with the point-to-point iteration required to resolve the Range-Doppler model equation set.

There are many geometric correction methods in terms of model used, including the physical model, the mathematic model and the collinearity equation model. With respect to the auxiliary data, there are two fundamental approaches: with control points and without control points. Numerous studies in the literature focus on one method or one condition, but lack a systematic analysis of model performance and usability. This paper compares and summaries the performances, including efficiency, robustness and precision, of four models applied in different terrain: the RD model, the RPC model, a revised polynomial model improved by this paper and the elevation derivation method. Different application requirements are considered, when deciding appropriate geometric correction models to achieve a balance of efficiency and precision.

## 2. Description of the Spaceborne SAR Geometric Correction Model

### 2.1. Range-Doppler Model

The Range-Doppler model is based on rigorous SAR imaging geometry principles. The location of an arbitrary pixel in a digital image is determined by the intersection of the centroid of the radar beam with the planet surface [1].

The core of the RD model is the three fundamental functions: the Earth Model Equation, the SAR Doppler Equation and the SAR Range Equation.

A. Earth model equation

(1) $$\frac{X_{t}^{2}+Y_{t}^{2}}{{\left(R_{e}+h\right)}^{2}}+\frac{Z_{t}^{2}}{R_{P}^{2}}=1$$

where $\left(\begin{array}{ccc} X_{t} & Y_{t} & Z_{t} \end{array}\right)$ is the target position vector, $R_{e}$ is the mean equatorial radius and $R_{P}=(1-f)R_{e}$ is the polar radius, f is a flattening factor and h is the target height relative to the Earth model.

B. SAR Range Equation

The SAR range equation defines the distance from a sensor to a target. The slant range from the sensor to the pixel is defined by the equation:

(2) $$R(i,j)=\left|\vec{P_{S}(t_{ij})}-\vec{P_{T}}\right|=\frac{c\tau }{2}$$

where i is the azimuth number, j is the range number, and $t_{ij}$ is the time that the radar beam center intersects the target. $\vec{P_{S}}$ and $\vec{P_{T}}$ are the space and target position vectors, respectively, c is the light speed, and τ is the time delay.

C. SAR Doppler Equation

The SAR Doppler equation defining the plane of the centroid is given by

(3) $$f_{d}=-\frac{2}{\lambda }\frac{\left(\vec{P_{S}}-\vec{P_{T}})(\vec{V_{S}}-\vec{V_{T}}\right)}{R}$$

where λ is the radar wavelength, R is the sensor to target slant range, $f_{d}$ is the Doppler frequency associated with the return echo data, $\vec{P_{S}}$ is the spacecraft position vector, $\vec{P_{T}}$ is the target position vector, $\vec{V_{S}}$ is the spacecraft velocity vector and $\vec{V_{T}}$ is the target velocity vector.

In terms of the model itself, the RD model is precise. The only errors inherent in this approach result from the deviation of the true geoid from the model and the uncertainty in the spacecraft ephemeris [1]. With the generation of more accurate spacecraft ephemeris data and maintaining careful controls on any variation in the delay of the pulse sampling window, the location accuracy could be improved [1,2]. Therefore the RD model is the fundamental model and has optimum performance.

### 2.2. Rational Polynomial Coefficients Model

The rational polynomial coefficients model [5] uses the ratios of polynomials connecting target geographic coordinates $\left(D_{Latitude},D_{Longitude},D_{Height}\right)$ with corresponding pixel coordinates $\left(d_{sample},d_{line}\right)$. To strengthen the stability of solution, coordinates need to be scaled between −1 and 1. For an image, the following expression [8] is defined:

(4) $$Y=\frac{Num_{L}\left(P,L,H\right)}{Den_{L}\left(P,L,H\right)}$$

(5) $$X=\frac{Num_{s}\left(P,L,H\right)}{Den_{s}\left(P,L,H\right)}$$

where

(6) $$\begin{array}{r} Num_{L}\left(P,L,H\right)=a_{1}+a_{2}L+a_{3}P+a_{4}H+a_{5}LP+a_{6}LH+a_{7}PH+a_{8}L^{2}+a_{9}P^{2}+a_{10}H^{2}+a_{11}PLH+\\ a_{12}L^{3}+a_{13}LP^{2}+a_{14}LH^{2}+a_{15}L^{2}P+a_{16}P^{3}+a_{17}PH^{2}+a_{18}L^{2}H+a_{19}P^{2}H+a_{20}H^{3}\\ Den_{L}\left(P,L,H\right)=b_{1}+b_{2}L+b_{3}P+b_{4}H+b_{5}LP+b_{6}LH+b_{7}PH+b_{8}L^{2}+b_{9}P^{2}+b_{10}H^{2}+b_{11}PLH+\\ b_{12}L^{3}+b_{13}LP^{2}+b_{14}LH^{2}+b_{15}L^{2}P+b_{16}P^{3}+b_{17}PH^{2}+b_{18}L^{2}H+b_{19}P^{2}H+b_{20}H^{3}\\ Num_{S}\left(P,L,H\right)=c_{1}+c_{2}L+c_{3}P+c_{4}H+c_{5}LP+c_{6}LH+c_{7}PH+c_{8}L^{2}+c_{9}P^{2}+c_{10}H^{2}+c_{11}PLH+\\ c_{12}L^{3}+c_{13}LP^{2}+c_{14}LH^{2}+c_{15}L^{2}P+c_{16}P^{3}+c_{17}PH^{2}+c_{18}L^{2}H+c_{19}P^{2}H+c_{20}H^{3}\\ Den_{S}\left(P,L,H\right)=d_{1}+b_{2}L+b_{3}P+b_{4}H+b_{5}LP+b_{6}LH+b_{7}PH+b_{8}L^{2}+b_{9}P^{2}+b_{10}H^{2}+b_{11}PLH+\\ b_{12}L^{3}+b_{13}LP^{2}+b_{14}LH^{2}+b_{15}L^{2}P+b_{16}P^{3}+b_{17}PH^{2}+b_{18}L^{2}H+b_{19}P^{2}H+b_{20}H^{3} \end{array}$$

where *a_i_*(i = 1,2,...,20), *b_i_*(i = 1,2,...,20), *c_i_*(i = 1,2,...,20), *d_i_*(i = 1,2,...,20) are the undetermined coefficients of the polynomial, $b_{1}$ and $d_{1}$ are always equal to 1, (P,L,H) is the normalised geographic coordinate and (X,Y) is the normalised image coordinate.

### 2.3. Revised Polynomial Model

The polynomial approach is easy to implement for a transformation based on two planes without considering the influence of undulating terrain; its utility is restricted to flat terrain area SAR images. Furthermore, sufficient ground control points (GCPs) are needed to solve the equation. Its fundamental idea is that the distortion of a remote sensing picture is a result of the translation, zoom, rotation, affinity, twisting, winding and combinations of more basic deformations [15]. The orthorectification of SAR images is based on the transformation from an image plane (x,y) to an earth plane (X,Y). The third-order polynomial equation is given by:

(7) $$\begin{array}{c} X=a_{0}+a_{1}x+a_{2}y+a_{3}x^{2}+a_{4}xy+a_{5}y^{2}+a_{6}x^{3}+a_{7}x^{2}y+a_{8}xy^{2}+a_{9}y^{3}\\ Y=b_{0}+b_{1}x+b_{2}y+b_{3}x^{2}+b_{4}xy+b_{5}y^{2}+b_{6}x^{3}+b_{7}x^{2}y+b_{8}xy^{2}+b_{9}y^{3} \end{array}$$

where (x,y) is the image coordinate of a pixel, (X,Y) is the geographic coordinate of the pixel and *a_i_*(i = 0,1,...,9), b_i_(i = 0,1,...,9) are the undetermined coefficients of the polynomial.

The polynomial model is not suitable for the geometric correction of SAR images of mountainous terrain because its simple transform is based on two planes without consideration of the influence of undulating terrain. This paper aims to correct this defect by providing a revised polynomial (PM) model based on elevation, given by the following equation:

(8) $$\begin{array}{c} X=a_{0}+a_{1}B+a_{2}L+a_{3}B^{2}+a_{4}BL+a_{5}L^{2}+a_{6}H+a_{7}H^{2}\\ Y=b_{0}+b_{1}B+b_{2}L+b_{3}B^{2}+b_{4}BL+b_{5}L^{2}+b_{6}H+b_{7}H^{2} \end{array}$$

where (X,Y) is the image coordinate of a pixel, *B*, *L* and *H* are the latitude, longitude and height of (B,L,H), the geographic coordinate of the pixel and *a_i_*(i = 0,1,...,7), b_i_(i = 0,1,...,7) are the undetermined coefficients of the polynomial.

### 2.4. Elevation Derivation Model

The elevation derivation model determines the original image coordinates corresponding to the well-distributed geodetic coordinate grid points. The method only performs the Range-Doppler equation iterative solution on the sampled SAR grid points; the remaining pixels are directly calculated by polynomials, which remarkably reduces the number of iterations and enhances the efficiency [16].

First, digital elevation modal (DEM) data related to the SAR image were coarse sampled at the intervals of k×l along the latitude and longitude axes, using n height level ($h_{i},i=1,2,...,n$), thus deriving n groups of new DEM data. Then, the n groups of coarse sampled DEM data were geolocated using the RD model, and the SAR image pixel positions ($P_{Ti}=(a_{Ti},r_{Ti}),i=1,2,...,n$) of the data were obtained. Two m-second (m < n) polynomials ($p_{a_{T}(x_{s},y_{s})}\left(h\right)$ and $p_{r_{T}(x_{s},y_{s})}\left(h\right)$) with elevation h were then calculated at each pixel position $\left(x_{s},y_{s}\right)$ of the coarse sampled DEM data. Then the polynomials $p_{a_{T}(x,y)}^{\prime }\left(h\right)$ and $p_{r_{T}(x,y)}^{\prime }\left(h\right)$ were calculated at each pixel position (x,y) of the original DEM data by using bilinear interpolation on the above polynomials:

(9) $$p_{a_{T}(x,y)}^{\prime }\left(h\right)=bilinear\left(\left[\begin{array}{cc} p_{a_{T}(\mathrm{floor}(x/k),\mathrm{floor}(y/l))}\;\left(h\right) & p_{a_{T}(\mathrm{floor}(x/k),\mathrm{floor}(y/l)+1)}\;\left(h\right)\\ p_{a_{T}(\mathrm{floor}(x/k)+1,\mathrm{floor}(y/l))}\;\left(h\right) & p_{a_{T}(\mathrm{floor}(x/k)+1,\mathrm{floor}(y/l)+1)}\;\left(h\right) \end{array}\right]\right)$$

(10) $$p_{r_{T}(x,y)}^{\prime }\left(h\right)=bilinear\left(\left[\begin{array}{cc} p_{a_{T}(\mathrm{floor}(x/k),\mathrm{floor}(y/l))}\;\left(h\right) & p_{a_{T}(\mathrm{floor}(x/k),\mathrm{floor}(y/l)+1)}\;\left(h\right)\\ p_{a_{T}(\mathrm{floor}(x/k)+1,\mathrm{floor}(y/l))}\;\left(h\right) & p_{a_{T}(\mathrm{floor}(x/k)+1,\mathrm{floor}(y/l)+1)}\;\left(h\right) \end{array}\right]\right)$$

where bilinear(•) represents bilinear interpolation operation and floor(•) represents approaching negative infinity.

Finally, by taking the elevation data $h_{\left(x,y\right)}$ of each pixel position (x,y) in the original DEM, substituting into $p_{a_{T}(x_{s},y_{s})}^{\prime }\left(h\right)$ and $p_{r_{T}(x_{s},y_{s})}^{\prime }\left(h\right)$ respectively, and calculating the position in the SAR image corresponding to each pixel of the original DEM, the geocoded image can be acquired by a SAR image resample.

### 2.5. Performance Prediction of the Models

Generally speaking, the model with a simpler configure can be more efficient, whereas the accuracy is lower and vice versa. The RD model consisting of a group of nonlinear equations describes the earth model, Doppler frequency variety and slant range calculation. Hence it is complicated and precise. The only errors inherent in this approach result from the deviation of the true geoid from the model and the uncertainty in the spacecraft ephemeris [1]. The RPC model with dozens of model parameters uses the ratios of polynomials to connect target geographic coordinates with corresponding pixel coordinates. However, once the parameters of the models are determined, it remains unchanged through the image processing. Therefore, the RPC model is not very adaptive. The PM model is similar to the simplification of the RPC model, so its accuracy can hardly exceed that of the RPC model. Nevertheless, the efficiency is improved significantly, which is a positive point. In the EDM model, the geographic coordinates of the coarse grid points were firstly calculated by the RD model, and then the relationship between the elevation of an image point and the two-dimensional geographic coordinates can be described by two polynomials, respectively. Finally, the polynomial coefficients of the geographic grid points were interpolated. It can be found that the polynomial coefficients of each point are different. Therefore, high-precision and fast geolocation can be achieved.

## 3. Experiment and Analysis

### 3.1. Experiment Data and Explanation

**Figure 1:**
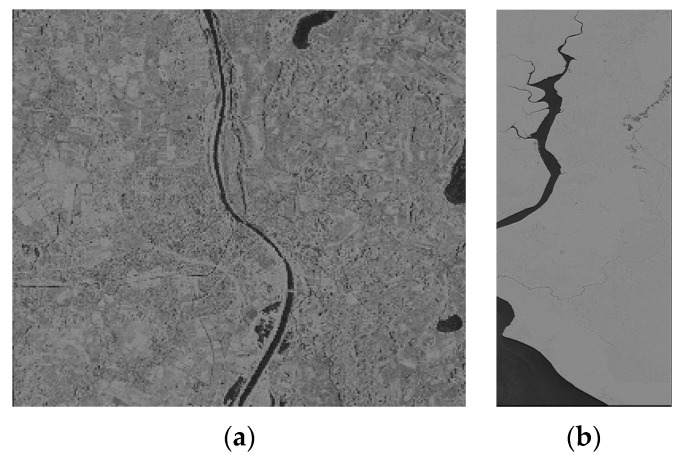
Flat terrain SAR images before geometric correction, (**a**) T1 SAR image and (**b**) T3 SAR image.

**Figure 2:**
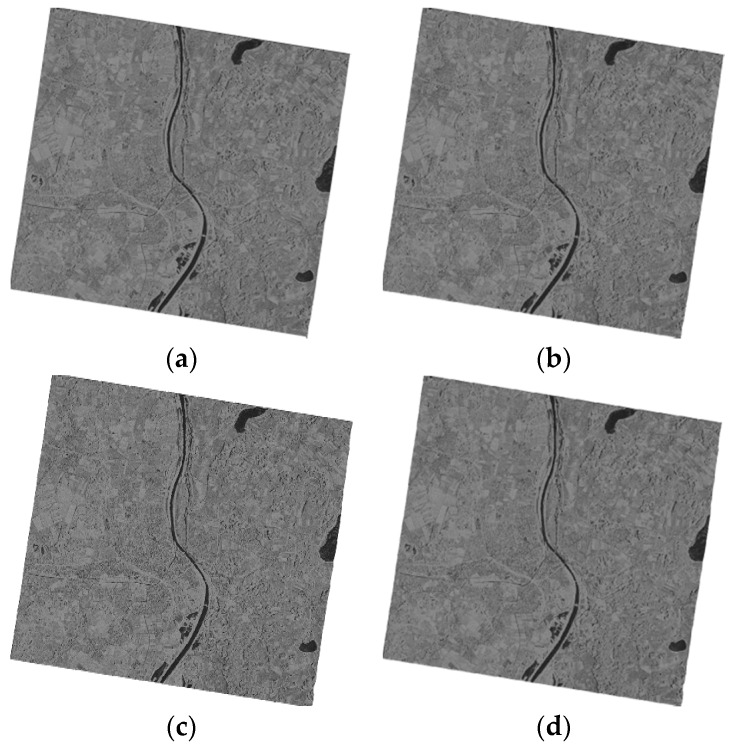
Geometric correction results for the T1 SAR image on flat terrain, (**a**) The RD model; (**b**) The RPC model; (**c**) The PM model; and (**d**) The EDM model.

**Figure 3:**
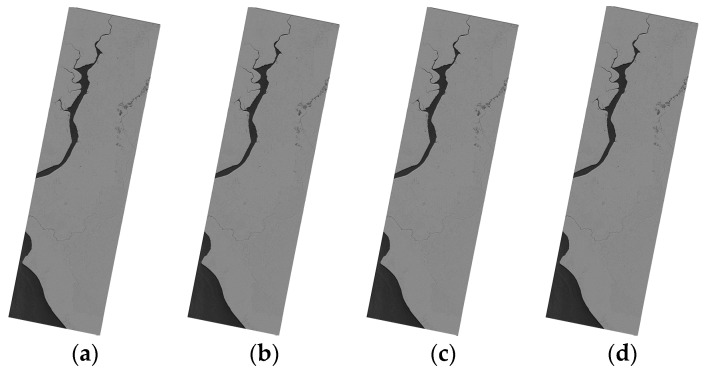
Geometric correction results for the T3 SAR image on flat terrain, (**a**) The RD model; (**b**) The RPC model; (**c**) The PM model and (**d**) The EDM model.

**Figure 4:**
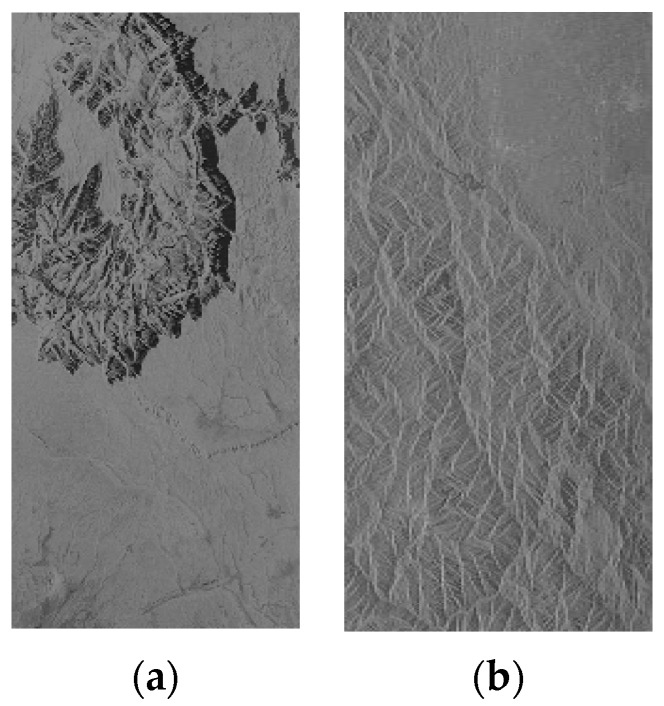
Mountain terrain SAR images before geometric correction, (**a**) T2 SAR image and (**b**) P1 SAR image, with the image size adjusted for a clear look.

We used two groups of spaceborne SAR images to conduct the experiment. The first group data consists of two SAR images from TerraSAR-X and one image from Envisat-ASAR, named T1, T2 and E1 respectively. The T1 SAR image is of flat terrain (Figure 1a) and the T2 SAR image is of undulating terrain (Figure 1b). The geolocation results for the T1 and T2 SAR images are shown in Figure 2 and Figure 3 respectively. The E1 SAR image dataset has a scene size of 103 km × 632 km, large coverage areas and complicated surfaces. Information details for the datasets are shown in Table 1. There are also another three SAR images including one TerraSAR-X image and two ALOS-PALSAR images in the second group data, named T3, P1 and P2 respectively. The T3 SAR image is of flat terrain (Figure 4a) and the P1 SAR image (Figure 4b) is of undulating terrain. The P2 SAR image dataset has a scene size of 414 km × 91 km, large coverage areas and complicated surfaces. Information and details for the datasets are shown in Table 2. In addition, the use of different sensors has no effect on the results and the different sensors are only used due to data availability.

Terrain classification categories of topographic are that the terrain classification is according to the slope and difference in elevation of the greater part of the image. When the difference in elevation is controversial with the slope, it is the slope that acts as the criterion [18]. The reference [19] shows that the optimal combination of classification indexes includes elevation, total accumulation curvature, variability of slope, hill shade, variance coefficient in elevation, contour line density, and range. The importance arrangement of classification indexes is: elevation > variance coefficient in elevation > contour line density > range > total accumulation curvature > variability of slope > hill shade. The terrain classification categories always include flat ground, hills, mountainous region and high mountain region. In this paper, the flat terrain means its altitude range is below 800 m and the difference in elevation less than 500 m as well as a slope less than 25°, and the altitudes range of mountainous terrain is always over 800 m and the difference in elevation greater than 500 m as well as a slope larger than 25°. The large scene was defined that the length of the scene size is over 100 km. The importance arrangement of classification indexes is: slope > difference in elevation > elevation. The specification of terrain classification categories was shown in Table 3.

The selection of the control points and check points was random based on the geometric correction image grid, in which the former is used for equation solution and the latter means the point where evaluation accuracy was determined. To evaluate the performance of the models, we calculated the maximum error and the root mean square error (RMSE) in azimuth and range directions and in the plane. Additionally, the computational run time was tested to quantify model efficiency. The comparison was made with the RD method, in which the RD data was thought the most accurate [9].

### 3.2. Result of Experiment and Analysis of Performance

#### 3.2.1. The Geometric Correction Results of SAR Images on Flat Terrain

The flat terrain SAR images (T1 and T3) before geometric correction are showed in Figure 1. The geolocation results for the four models are shown in Figure 2 and Figure 3. From Figure 2 and Figure 3, the geolocation results seems little different from each other, which illustrated that the models have the similar geometric correction results for SAR images on flat terrain.

Table 4 and Table 5 show the geolocation accuracy of control points and check points of the two flat terrain SAR images, respectively. In the tables, RD represents the RD model, RPC represents the RPC model, PM represents the revised polynomial model and EDM represents the elevation derivation model. In the Table 5, the geolocation results of T1 and T3 SAR images both showed that the precision and efficiency of RPC model are better than those of the EDM model for the geometric correction of the flat terrain SAR images. The efficiency of RPC model is 2.88 times of the EDM model. The geolocation precision of the PM model is better than 1 pixel for both the azimuth and range, and at the same time, its consumed time is approximately 50% and 20% of the RPC model and EDM model respectively, which illustrates that the PM model has a good rectification capacity for flat terrain.

#### 3.2.2. The Geometric Correction Results of SAR Images on Mountain Terrain

**Figure 5:**
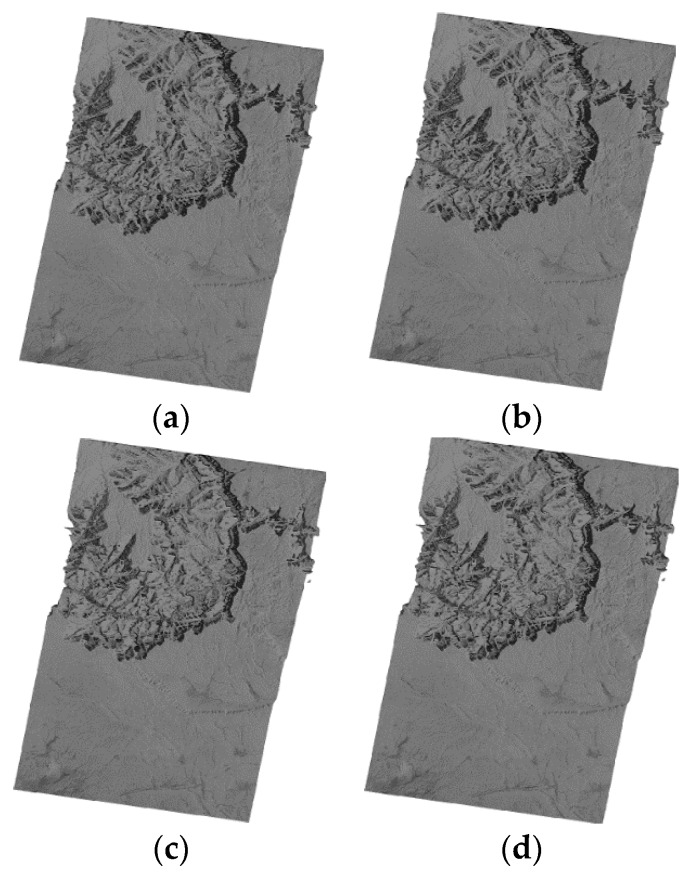
Geometric correction results for the T2 SAR image on mountain terrain, (**a**) the RD model; (**b**) the RPC model; (**c**) the PM model; and (**d**) the EDM model.

**Figure 6:**
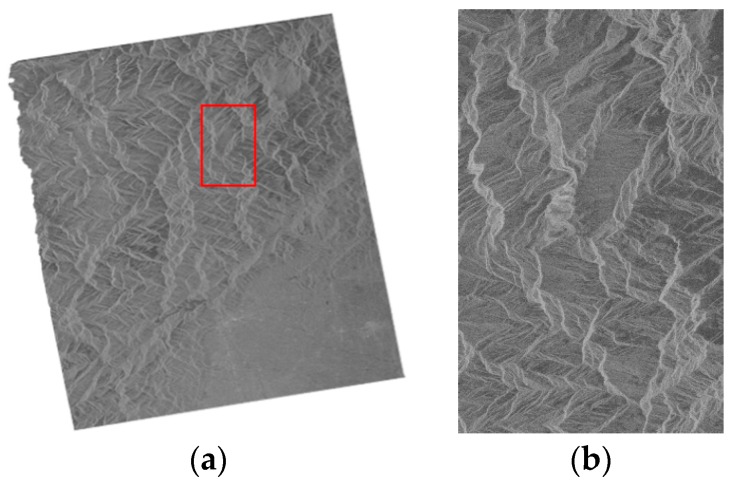

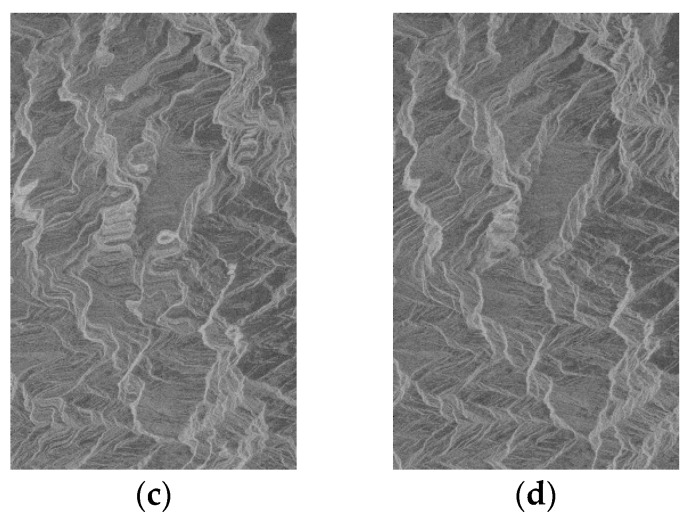
Geometric correction results for the P1 SAR image on mountain terrain, (**a**) the RD model; (**b**) the RPC model for the red rectangle; (**c**) the PM model for the red rectangle; and (**d**) the EDM model for the red rectangle.

The mountain terrain SAR images (T2 and P1) before geometric correction are shown in Figure 4. The geometric correction results of the mountain terrain SAR images are shown in Figure 5 and Figure 6. In Figure 6b,c, the difference between the geometric correction results of the RPC model and the PM model is clearly showed in the red rectangle. In addition, the difference between the RPC model and EDM model can hardly be distinguished, which can be further referred to the quantitative results.

Both the RPC model and the EDM model have high precision and efficiency as seen in Table 6 and Table 7. However, the RPC model has higher efficiency and the EDM model has higher precision. The plane error RMSE of T2 image is 0.0124 pixels and the range error RMSE of T2 is 0.00945 pixels of the RPC model, while the plane error RMSE of T2 image is 0.00369 pixels and the range error RMSE of T2 is 0.00395 pixels of the EDM model. The range error and plane error of the RPC model are obviously worse than the EDM model for the geometric correction of mountain terrain images. However, the efficiency of the RPC model is nearly twice that of the EDM model. The PM model has the highest efficiency and the geolocation precision is the worst. Moreover, the azimuth location accuracy is better for the PM model, but the range location accuracy is lower, confirming that the PM model cannot properly correct the location errors resulting from undulating terrain.

#### 3.2.3. The Geometric Correction Results of SAR Images of the Large Area

**Figure 7:**
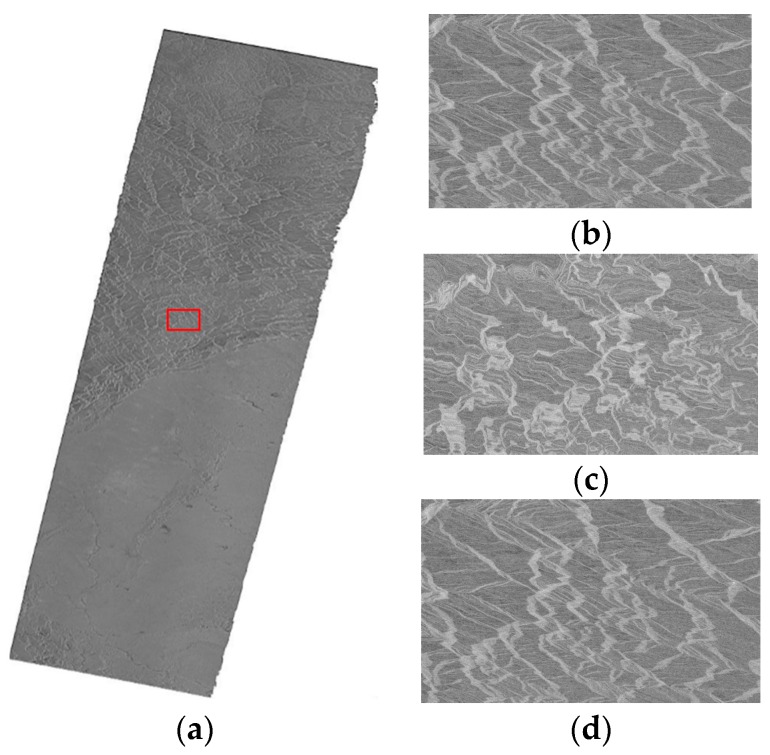
Geometric correction results for the E1 SAR image of the large area, (**a**) the RPC model; (**b**) the RPC model for the red rectangle; (**c**) the PM model for the red rectangle; and (**d**) the EDM model for the red rectangle.

**Figure 8:**
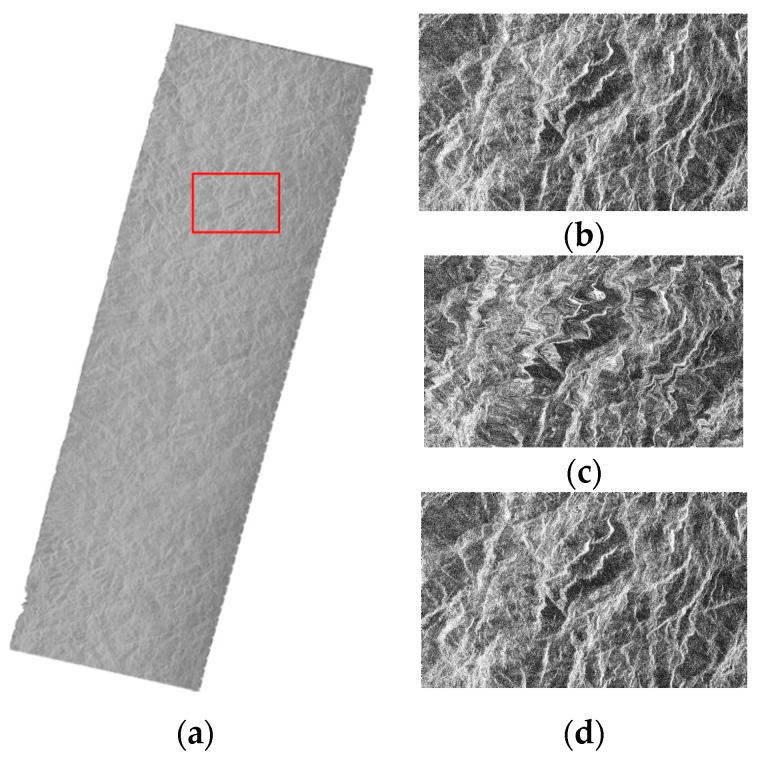
Geometric correction results for the P2 SAR image of the large area, (**a**) The RPC model; (**b**) The RPC model for the red rectangle; (**c**) The PM model for the red rectangle; and (**d**) The EDM model for the red rectangle. In order to make the difference visually, the figure (**b**–**d**) were processed with image enhancement.

The correction results for the large scene SAR images are shown in Figure 7 and Figure 8, including results for the RPC model, PM model and EDM model. It can be seen that the location accuracy of the PM model decreases greatly for the rectification of the large SAR image, which can be confirmed from Figure 7c and Figure 8c, in which the mountain skeleton is blurred, especially the part marked with red rectangle. The quantitative results are showed in Table 8 and Table 9. For the RPC model, the azimuth location accuracy is below one pixel, but the range location accuracy is far worse. In Table 9, the plane error RMSE of the E1 image is 3.68 pixels and the plane error RMSE of P2 is 0.467 pixels of the RPC model, while the plane error RMSE of E1 image is 0.55 pixels and the plane error RMSE of P2 is 0.0384 pixels of the EDM model. The correction results for the EDM model can still maintain better accuracy and lower efficiency compared with the other two models. The efficiency of the RPC model even achieves three times that of the EDM model for the geometric correction of large scene SAR images. It is reasonable for the large scene to require more processing time. Therefore, the EDM model is suitable for the geometric correction of large SAR images when high geolocation precision is required.

#### 3.2.4. Discussion and Solution Table

The following conclusions can be made: First, the primary reason for the plane location error is that the range location error is obviously higher than the azimuth for all models. Second, the PM model had the best efficiency; however, its accuracy was the worst. Although an error of only 0.03 pixels was produced on the flat terrain image, its maximum geolocation error reached 5.65 pixels on the mountain terrain image; the plane location error was more than 29 pixels, especially on the large scene image, which cannot meet the precision required for conventional applications. Third, the accuracies of the RPC model and the EDM model were better than that of the PM model whatever the maximum or mean square error; between them, the RPC model had higher efficiency, whereas the EDM model was more robust. When they were used for the mountain terrain and large scene images, the precision of the RPC model declined dramatically; the maximum error reached 36 pixels and the RMSE is 3.6 pixels for the large scene image; however, the plane location accuracy of the EDM model could still be better than 1 pixel.

Based on the above experiments, aiming at different terrain and large scene SAR images, a solution can be made to choose an optional model which can be fulfilled with the requirement of a user for the geometric correction of a spaceborne SAR image, shown in Table 10. The “×” means the model was not recommended due to having the lowest efficiency or the worst accuracy. “Recommended” means the model is recommended for its high precision and “Acceptable” means the model is available when its precision meets the demand of the user as well as its high efficiency. For example, in terms of the geometric correction of mountain terrain SAR image, if high precision is required, the EDM model is recommended. If the high precision is not required, the RPC model is acceptable.

## 4. Conclusions

The RD model is the standardized method of geometric correction but is not suitable for real time applications because of large computing times. The PM model is efficient but is the least precise. It was valuable for the flat terrain SAR image rectification of the polynomial model, for which the maximum error was 0.19 pixels and the plane error was 0.02 pixels. Compared with the RD model, the RPC model and the EDM model lost some precision but their efficiencies were greatly improved. Therefore, the two models have higher value for engineering applications.

The EDM model has stable precision regardless of terrain variation. Compared with the EDM model, the location error of the RPC model was slightly higher on the mountain terrain area but was much lower on the flat terrain area because the EDM model could better rectify the location errors resulting from the terrain on the undulating terrain area. On the flat terrain area, because of the smaller location error caused by terrain, the RPC model can precisely describe the relationship between geographic position and image coordinates. The PM model has potential because of its high efficiency; future work should be conducted on correcting the influence of elevation.

Every model has its own characteristics with respect to algorithm precision, efficiency and robustness, and an appropriate model can be selected based on the application requirements and the features of the SAR image. Considering their comprehensive advantages with respect to precision, efficiency and robustness, we propose to use the EDM model or RPC model for conventional applications. When the topography is complicated or the image is too large, the EDM model is recommended. The PM model can be used for flat terrain areas where there is no requirement for high precision. The users can consult Table 10 before their choosing a model.

## Figures and Tables

**Table 1:** Specification of experimental data of the first group.

Image Sensor	TerraSAR-X	TerraSAR-X	Envisat-ASAR
Acquisition Time	27 January 2008	21 March 2008	6 August 2008
Center Longitude (°)	12.14	131.02	104.39
Center Latitude (°)	47.68	–25.34	31.76
Image Size (azimuth × range)	8330 × 9504	15,328 × 32,686	155,688 × 5158
Pixel Size (m)	1.2 × 1.9	1.3 × 1.8	4.1 × 19.9
Altitude Range (m)	436–604	726–2793	48–4133
Topography	Flat	Mountain	Flat, Mountain

**Table 2:** Specification of experimental data of the second group.

Image Sensor	TerraSAR-X	ALOS-PLASAR	ALOS-PLASAR
Acquisition Time	13 March 2008	7 May 2008	12 April 2009
Center Longitude (°)	111.82	103.56	109.37
Center Latitude (°)	−2.72	31.20	29.17
Image Size (azimuth × range)	23,361 × 10,288	35,192 × 4761	101,938 × 4962
Pixel Size (m)	2.4×0.9	3.1×14.9	4.1 × 18.5
Altitude Range (m)	−2–113	468–5272	80–2557
Topography	Flat	Mountain	Flat, Mountain

**Table 3:** Specification of terrain classification categories.

Topography	Flat	Mountainous	Large Scene
Slope (°)	<25	>25	-
Difference in Elevation	<500	>500	-
Elevation	<800	>800	-
Scene Size (km)	-	-	>100

**Table 4:** Geolocation accuracy of control points on flat terrain.

Data	Models	Azimuth Error (Pixel)		Range Error (Pixel)		Plane Error (Pixel)	
		Max	RMSE	Max	RMSE	Max	RMSE
	RD	0	0	0	0	0	0
T1	RPC	7.91 × 10^−5^	1.86 × 10^−5^	9.12 × 10^−5^	3.49 × 10^−5^	1.00 × 10^−4^	4.57 × 10^−5^
	PM	4.73 × 10^−3^	1.76 × 10^−3^	1.90 × 10^−1^	2.53 × 10^−2^	1.90 × 10^−1^	2.55 × 10^−2^
	EDM	7.27 × 10^−12^	7.98 × 10^−13^	7.27 × 10^−12^	9.72 × 10^−13^	9.09 × 10^−12^	1.43 × 10^−12^
T3	RPC	9.01 × 10^−5^	2.22 × 10^−5^	1.85 × 10^−3^	6.70 × 10^−4^	1.85 × 10^−3^	6.75 × 10^−4^
	PM	3.66 × 10^−2^	7.15 × 10^−3^	2.44	5.16 × 10^−1^	2.44	5.16 × 10^−1^
	EDM	2.54 × 10^−11^	2.69 × 10^−12^	1.27 × 10^−11^	1.36 × 10^−12^	2.54 × 10^−11^	3.39 × 10^−12^

**Table 5:** Geolocation accuracy of check points on flat terrain.

Data	Models	Azimuth Error (Pixel)		Range Error (Pixel)		Plane Error (Pixel)		Run Time (s)
		Max	RMSE	Max	RMSE	Max	RMSE	
	RD	0	0	0	0	0	0	>14,400
T1	RPC	2.40 × 10^−4^	5.63 × 10^−5^	4.90 × 10^−3^	9.19 × 10^−5^	4.90 × 10^−3^	1.20 × 10^−4^	24.47
	PM	4.73 × 10^−3^	1.90 × 10^−3^	2.83 × 10^−1^	3.45 × 10^−2^	2.83 × 10^−1^	3.48 × 10^−2^	15.11
	EDM	7.90 × 10^−4^	5.30 × 10^−4^	4.39 × 10^−3^	2.90 × 10^−3^	4.45 × 10^−3^	2.95 × 10^−3^	64.75
T3	RPC	2.56 × 10^−4^	6.29 × 10^−5^	7.40 × 10^−3^	1.40 × 10^−3^	7.40 × 10^−3^	1.41 × 10^−3^	111.65
	PM	1.29 × 10^−2^	6.91 × 10^−3^	8.75 × 10^−1^	4.75 × 10^−1^	8.75 × 10^−1^	4.75 × 10^−1^	55.52
	EDM	4.95 × 10^−5^	2.49 × 10^−5^	1.65 × 10^−2^	1.10 × 10^−2^	1.65 × 10^−2^	1.10 × 10^−2^	316.55

**Table 6:** Geolocation accuracy of control points on mountain terrain.

Data	Models	Azimuth Error (Pixel)		Range Error (Pixel)		Plane Error (Pixel)	
		Max	RMSE	Max	RMSE	Max	RMSE
	RD	0	0	0	0	0	0
T2	RPC	8.16 × 10^−3^	2.07 × 10^−3^	1.49 × 10^−2^	4.96 × 10^−3^	1.58 × 10^−2^	6.08 × 10^−3^
	PM	4.14 × 10^−2^	9.23 × 10^−3^	3.31	5.95 × 10^−1^	3.31	5.96 × 10^−1^
	EDM	2.91 × 10^−11^	3.09 × 10^−12^	1.45 × 10^−11^	1.65 × 10^−12^	3.05 × 10^−11^	3.95 × 10^−12^
P1	RPC	5.03 × 10^−3^	1.55 × 10^−3^	1.37 × 10^−2^	5.53 × 10^−3^	1.37 × 10^−2^	5.75 × 10^−3^
	PM	2.18 × 10^−1^	5.56 × 10^−2^	2.41	5.63 × 10^−1^	2.42	5.66 × 10^−1^
	EDM	2.91 × 10^−11^	5.12 × 10^−12^	3.63 × 10^−12^	7.20 × 10^−13^	2.91 × 10^−11^	5.16 × 10^−12^

**Table 7:** Geolocation accuracy of check points on mountain terrain.

Data	Models	Azimuth Error (Pixel)		Range Error (Pixel)		Plane Error (Pixel)		Run Time (s)
		Max	RMSE	Max	RMSE	Max	RMSE	
	RD	0	0	0	0	0	0	>14,400
T2	RPC	2.52 × 10^−2^	6.04 × 10^−3^	5.23 × 10^−2^	9.45 × 10^−3^	5.27 × 10^−2^	1.24 × 10^−2^	167.15
	PM	5.83 × 10^−2^	8.74 × 10^−2^	5.65	7.87 × 10^−1^	5.65	7.88 × 10^−1^	146.80
	EDM	5.10 × 10^−4^	3.40 × 10^−3^	5.92 × 10^−3^	3.95 × 10^−3^	5.94 × 10^−3^	3.96 × 10^−3^	379.69
P1	RPC	5.62 × 10^−3^	2.47 × 10^−3^	1.04 × 10^−1^	2.62 × 10^−2^	1.05 × 10^−1^	2.63 × 10^−2^	15.37
	PM	8.41 × 10^−1^	1.93 × 10^−1^	5.13	1.42	5.20	1.44	11.26
	EDM	5.25 × 10^−3^	3.68 × 10^−3^	8.06 × 10^−3^	5.65 × 10^−3^	9.62 × 10^−3^	6.74 × 10^−3^	51.37

**Table 8:** Geolocation accuracy of control points in a large area.

Data	Models	Azimuth Error (Pixel)		Range Error (Pixel)		Plane Error (Pixel)	
		Max	RMSE	Max	RMSE	Max	RMSE
	RD	0	0	0	0	0	0
E1	RPC	1.52 × 10^−1^	6.07	1.45	6.08	1.49	9.93 × 10^−1^
	PM	1.75	121.26	25.07	121.58	25.21	14.21
	EDM	1.52 × 10^−11^	5.45 × 10^−12^	5.19 × 10^−13^	8.73 × 10^−11^	1.54 × 10^−11^	8.73 × 10^−11^
P2	RPC	2.87 × 10^−2^	7.55 × 10^−2^	2.82 × 10^−1^	7.55 × 10^−1^	2.84 × 10^−1^	1.03 × 10^−1^
	PM	1.15	33.19	7.43	33.84	7.52	6.62
	EDM	1.44 × 10^−11^	5.45 × 10^−12^	9.22 × 10^−13^	7.29 × 10^−11^	1.45 × 10^−11^	7.27 × 10^−11^

**Table 9:** Geolocation accuracy of check points in a large area.

Data	Models	Azimuth Error (Pixel)		Range Error (Pixel)		Plane Error (Pixel)		Run Time (s)
		Max	RMSE	Max	RMSE	Max	RMSE	
	RD	0	0	0	0	0	0	>14,400
E1	RPC	0.92	0.21	36.62	3.64	36.62	3.68	195.05
	PM	12.31	2.26	150.60	29.41	150.80	29.59	112.63
	EDM	0.15	0.09	0.88	0.54	0.90	0.55	870.78
P2	RPC	9.47 × 10^−2^	3.31 × 10^−2^	2.70	4.66 × 10^−1^	2.70	4.67 × 10^−1^	87.99
	PM	6.58	1.54	33.27	8.45	33.92	8.59	35.25
	EDM	7.91 × 10^−3^	5.74 × 10^−3^	5.19 × 10^−2^	3.80 × 10^−2^	5.25 × 10^−2^	3.84 × 10^−2^	237.37

**Table 10:** Solution to choose the suitable model.

	*Image*	Flat Terrain	Mountain Terrain	Large Scene
		Slope < 25°, Elevation < 800 m	Slope > 25°, Elevation > 800 m	Scene Size > 100 km
*Models*		Difference in Elevation < 500 m	Difference in Elevation > 500 m	
RD		-	-	-
RPC		Recommended	Acceptable	Acceptable
PM		Acceptable	-	-
EDM		-	Recommended	Recommended
